# Supplementary material for: SUPPA2: fast, accurate, and uncertainty-aware differential splicing analysis across multiple conditions
Source: Genome Biol. 2018 Mar 23;19:40. doi: 10.1186/s13059-018-1417-1 (PMC5866513; doi:10.1186/s13059-018-1417-1)
Supplement: Supplementary file 1 — Figures S1–S6. (PDF 3939 kb) [file 13059_2018_1417_MOESM1_ESM.pdf]

Supplementary Figures

SUPPA2 provides fast, accurate, and uncertainty-aware differential splicing analysis across multiple conditions

Juan L. Trincado[1,*], Juan C. Entizne[2,*], Gerald Hysenaj[3], Babita Singh[1], Miha Skalic[1], David J. Elliott[3], Eduardo Eyras[1,4]

[1]Pompeu Fabra University, E08003, Barcelona, Spain

[2]University of Dundee, Invergowrie, Dundee DD2 5DA, UK.

[3]Institute of Genetic Medicine, Newcastle University, Central Parkway, Newcastle NE1 3BZ, UK.

[4]ICREA, E08010, Barcelona, Spain

* equal contribution

corresponding author: eduardo.eyras@upf.edu

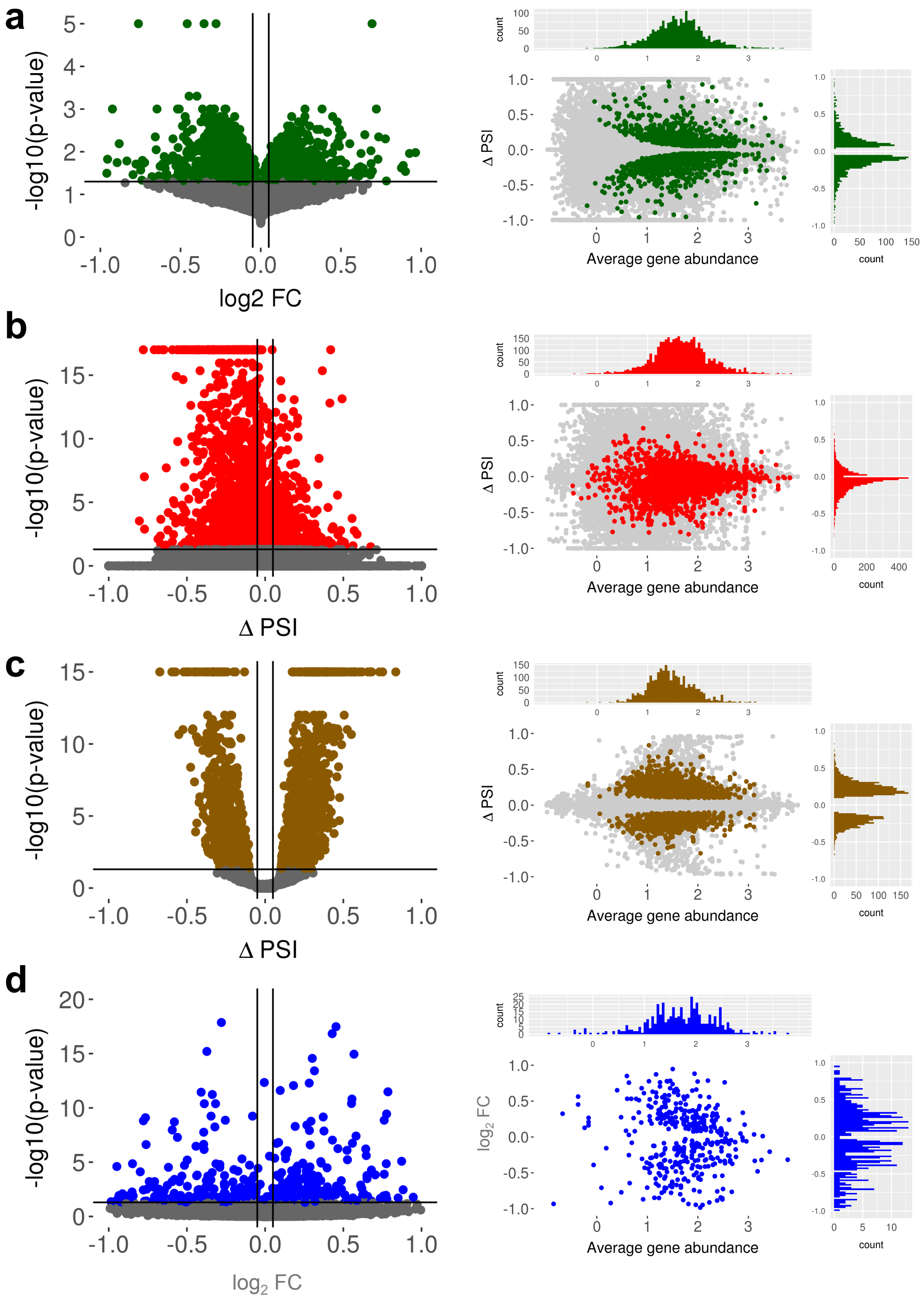

Figure S1. Comparison of volcano and MA-plots for differential splicing between methods. We built volcano plots (left panels) [1] and MA-like plots (right panels) [2] adapted for differential splicing results from **(a)** SUPPA2**, (b)** rMATS, **(c)** MAJIQ, and **(d)** DEXSeq, using the RNA-seq data from [3]. The volcano plots show the significance of each event (*y*-axis) as a function of the effect size (or signal change) (*x*-axis), which for SUPPA2, rMATS and MAJIQ is ΔPSI, and for DEXSeq is $\log_2$(fold-change). For MAJIQ we used as significance p-value = 1-P, where P is the posterior probability of |ΔPSI|>0.1 for a junction. The MA-like plots show the effect size (signal change) (*y*-axis) as a function of the average

gene expression (*x*-axis). As before, effect size for SUPPA2, rMATS and MAJIQ is given in terms of ΔPSI, and for DEXSeq is given by the $\log_2$(fold-change). To make these MA-plots comparable across methods, the *x*-axis shows the average gene expression in $\log_{10}$(TPM) scale for all methods. Average gene expression was calculated from the sum of abundances of transcripts per gene in TPM units. Transcript abundances in this case was estimated using Sailfish [4] on the Ensembl annotation (release 75). SUPPA2 significant events were calculated as explained in Methods. Additionally, for SUPPA2 and rMATS, only events of exon-cassette type (SE) are represented. For MAJIQ, we represented the junctions that were mapped to the cassette events from SUPPA2, using the junction with the largest posterior for |ΔPSI|>0.1 when this was duplicated in more than one LSV from MAJIQ. For DEXSeq we represented those exonic regions that matched exactly a regulated exon from the cassette events calculated from SUPPA2. The panels attached to each MA-plot display the distribution of the significant ΔPSI values along the x-axis (top panel) and along the y-axis (right panel). For SUPPA2 and rMATS the background (between-replicate) distributions was calculated subtracting the PSI values between replicates. For MAJIQ, we calculated the PSI values per sample and subtracted the values between replicates. For DEXSeq we could not calculate a similar background distribution, as it does not provide a value per sample. The cut-offs for significance used for these plots were corrected p-value < 0.05 for SUPPA2, FDR<0.05 for rMATS, posterior > 0.95 for MAJIQ and corrected p-value < 0.05 for DEXSeq.

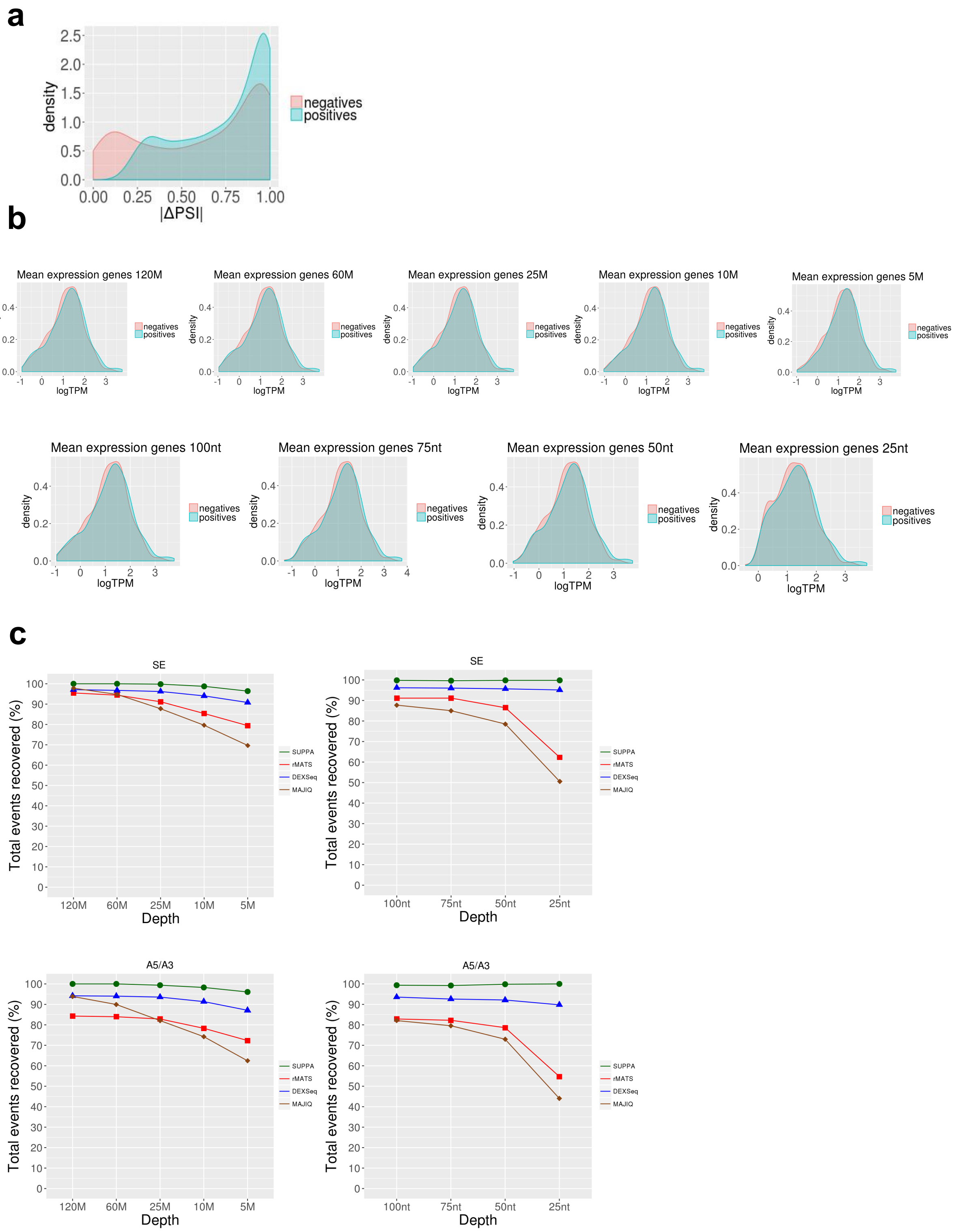

a
density
2.5
2.0
1.5
1.0
0.5
0.0
0.00 0.25 0.50 0.75 1.00
|ΔPSI|
negatives
positives
b
Mean expression genes 120M
Mean expression genes 60M
Mean expression genes 25M
Mean expression genes 10M
Mean expression genes 5M
Mean expression genes 100nt
Mean expression genes 75nt
Mean expression genes 50nt
Mean expression genes 25nt
density
logTPM
negatives
positives
c
SE
A5/A3
Total events recovered (%)
Depth
120M 60M 25M 10M 5M
100nt 75nt 50nt 25nt
SUPPA
rMATS
DEXSeq
MAJIQ

Figure S2. Simulation of differential splicing at different sequencing depths and read lengths. (a) For the simulation we considered genes with 2 transcripts isoforms only, and calculated the relative expression change between the two isoforms in absolute value: DeltaPSI = $|TPM_1-TPM_2|/(TPM_1+TPM_2)$. We considered the distribution of these values for the subset of genes that had a cassette event (SE), or an alternative 5' (A5) or 3' (A3) splice site event between the two transcript isoforms. Those cases such that DeltaPSI > 0.2 were used to sample the set of *positive genes*. We also sampled an equal number of genes with an SE event or an A5/A3 event, different from the previous set, from the entire distribution of DeltaPSI values, to define the *negative genes*. The plot shows the distributions of DeltaPSI values for the positive and negative sets. In **(b)** we show the distributions of gene expression values for the positive (blue) and negative (red) gene sets used for the benchmarking with simulated data, at different sequencing depths: 120, 60, 25, 10 and 5 million of paired-end (100nt long) reads (upper panels), and for different values of simulated read-lengths: 100nt, 75nt, 50nt, 25nt, all at a depth of 25M paired-end reads (lower panels). The x-axis indicates the gene expression in $\log_{10}$(TPM) units, where the TPM value per gene is obtained by adding up the (TPM) abundances from all transcripts in the gene. The distributions show all genes with SE and A5/A3 events used in the benchmarking. **(c)** Proportion of events that each method can detect (*y*-axis) from the positive and negative simulated events together at different sequencing depths (*x*-axis) (left panels) at a fixed length of 100nt, and at different read lengths (*x*-axis) (right panels) at fixed depth of 25M paired-end reads.

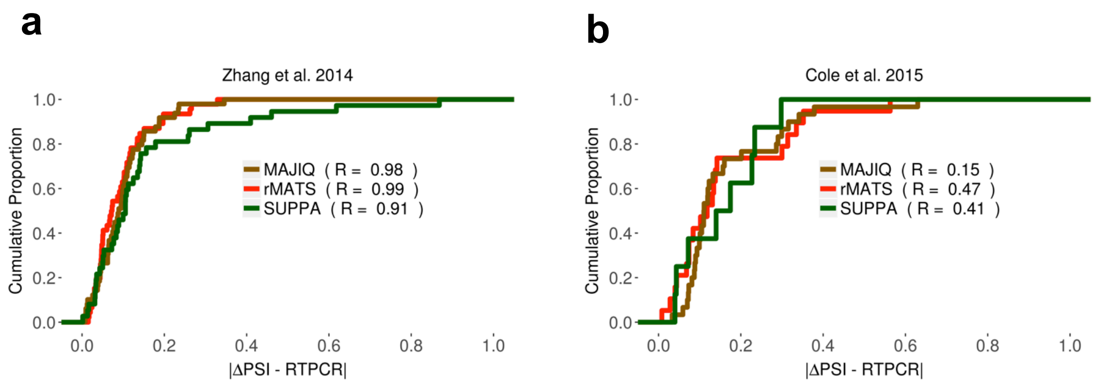

Figure S3. Comparison between predicted and experimental ΔPSI values. We compared the ΔPSI values estimated from RNA-Seq by SUPPA2, rMATS and MAJIQ with ΔPSI values obtained from RT-PCR experiments. For each RT-PCR set, we show the cumulative proportion of cases (*y*-axis) according to the absolute difference between the predicted and the experimental value (|ΔPSI – RTPCR|). Additionally, we give for each method the Pearson correlation R between predicted and experimental values. In **(a)** we show the comparison for 51 events using RT-PCR experiments in mouse cerebellum and liver [5] with ΔPSI estimates from RNA-Seq data from the same samples [6]. In this case, ΔPSI values from RT-PCR and RNA-seq were estimated from three experiments in each condition, each corresponding to three different stages of the circadian clock: 28, 40 and 52 hours. In **(b)** we show the comparison of ΔPSI values obtained from RT-PCR experiments in stimulated and unstimulated Jurkat cells [5] with the ΔPSI estimated from RNA-seq experiments from the same samples [7]. In this case we had two replicates for RNA-Seq per condition. As PSIs from RT-PCR were estimated without replication, in this case we only considered the 30 events (from the original 54) that had |ΔPSI|>0.05 from the RT-PCR [5].

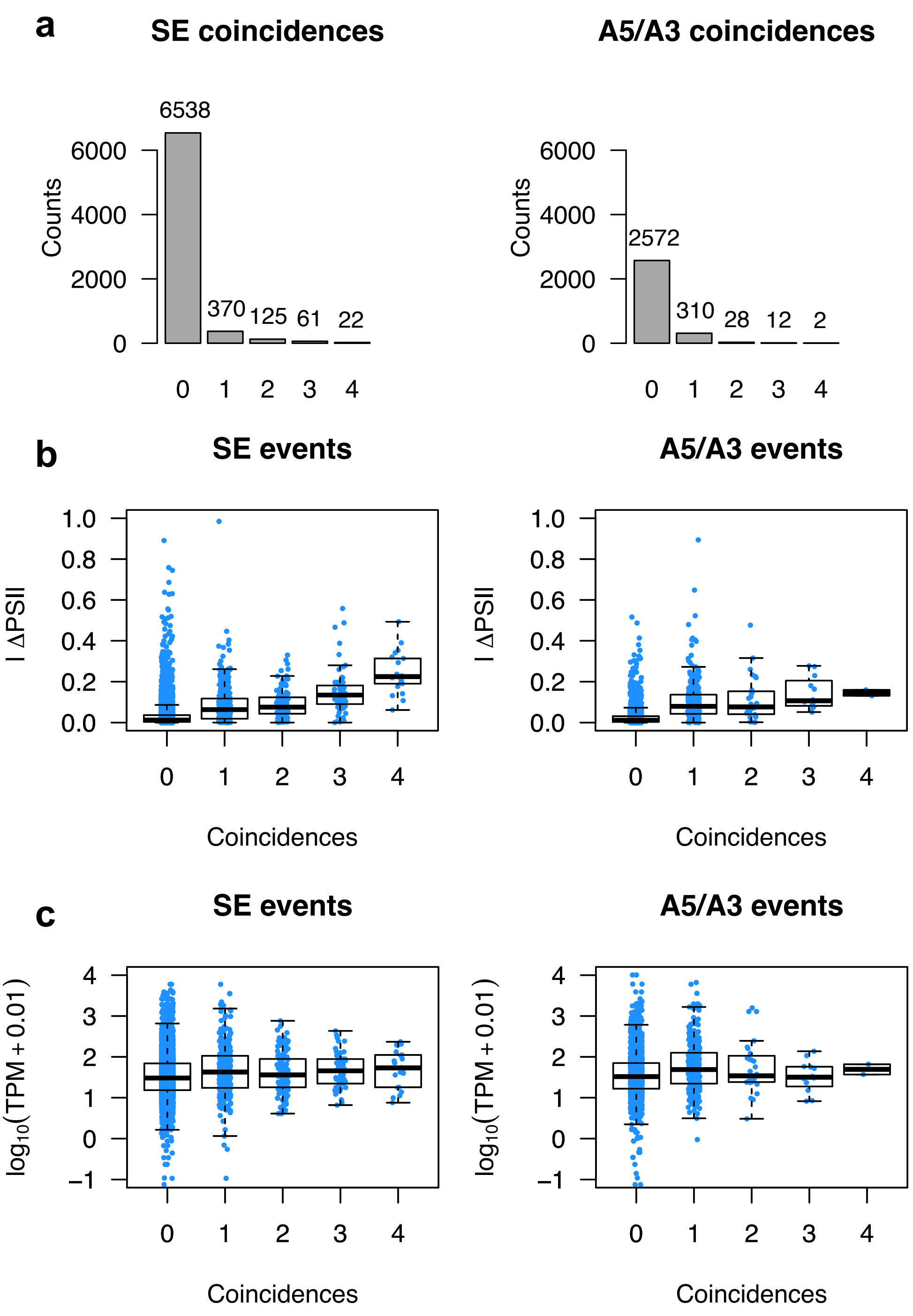
a
SE coincidences
A5/A3 coincidences
Counts
6538
370
125
61
22
2572
310
28
12
2
b
SE events
A5/A3 events
I ΔPSII
Coincidences
c
SE events
A5/A3 events
log10(TPM + 0.01)
Coincidences

Figure S4. Direct comparison of alternative splicing events from different methods. (a) We performed a direct comparison between alternative splicing events measured by different methods using events of type exon cassette (SE) and alternative 5' (A5) or 3' (A3) splice site. For SE events, these correspond to those common to SUPPA2 and rMATS, where the middle exon coincides with a DEXSeq exonic region, and where there is an inclusion junction compatible with the event from MAJIQ. For A5/A3 events, these correspond to event common to SUPPA2 and rMATS, with a variable region present in DEXSeq and with a compatible junction from MAJIQ. Further details are given in the Methods and Supplementary Methods. In the plot we show the number of events (*y*-axis), out of the 7116 SE events (left panel) and 2924 alternative A5/A3 events (right panel) that are detected by all 4 methods, and that are identified as significant by none, one, two, three or all four methods (*x*-axis). **(b)** Distribution of the **|ΔPSI|** values (*y*-axis) (measured by SUPPA2) for each of the sets defined in (a). **(c)** Distribution of gene expression values, in $\log_{10}$(TPM+0,01) units (*y*-axis), for each of the sets defined in (a). Gene expression was calculated by first adding up the TPM values for all transcripts in a gene, and then averaging the gene TPM values for the three replicates in the control condition.

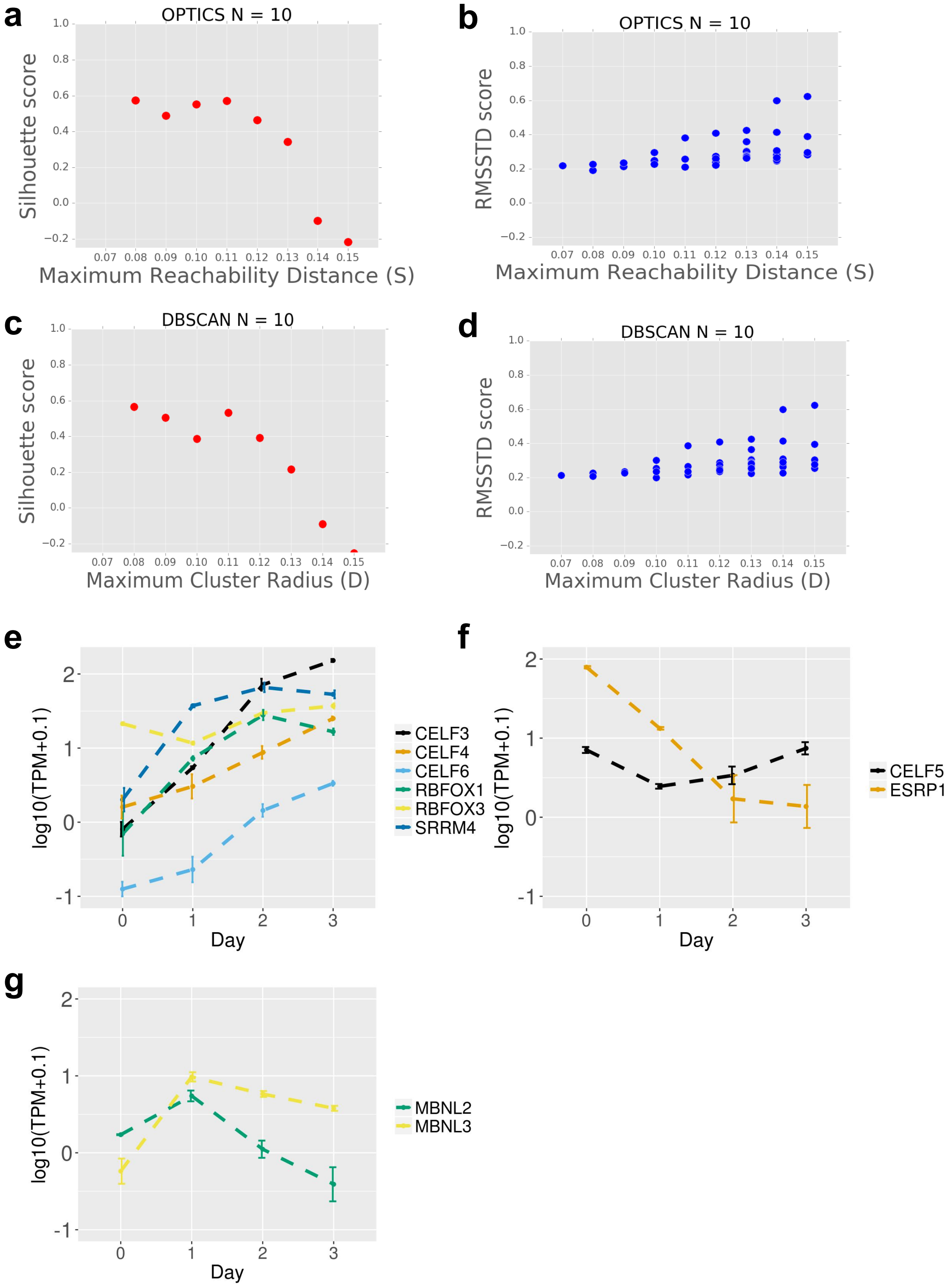

a
OPTICS N = 10
Silhouette score
Maximum Reachability Distance (S)
b
OPTICS N = 10
RMSSTD score
Maximum Reachability Distance (S)
c
DBSCAN N = 10
Silhouette score
Maximum Cluster Radius (D)
d
DBSCAN N = 10
RMSSTD score
Maximum Cluster Radius (D)
e
log10(TPM+0.1)
Day
CELF3
CELF4
CELF6
RBFOX1
RBFOX3
SRRM4
f
log10(TPM+0.1)
Day
CELF5
ESRP1
g
log10(TPM+0.1)
Day
MBNL2
MBNL3

Figure S5. Clustering of differentially spliced events across neuronal differentiation. The plots in **(a)**, **(b)**, **(c)** and **(d)** show the evaluation of the clustering results using two methods (OPTICS and DBSCAN) for differentially spliced cassette-exon events across 4 differentiation steps of human iPS cells differentiated into bipolar neurons [8]. Density-based clustering was performed for the 2780 cassette-exons events that changed splicing significantly in at least one comparison between any two adjacent steps. Clusters were evaluated for different values (*x*-axis) of the maximum reachability distance (S) for OPTICS [9] ((a) and (b)) and of the maximum (Euclidean) distance (D) to consider two events to be members of the same cluster (maximum cluster radius) for DBSCAN [10] ((c) and (d)). Quality of the clustering was evaluated with SUPPA2 by measuring the silhouette score (y axis) ((a) and (c)) and the root mean square standard deviation (RMSSTD) (y axis) ((b) and (d)) per cluster for the different S and D values (x axis) for each method. We also show in **(e)**, **(f)** and **(g)** the patterns of expression across the 4 differentiation days for the splicing factors that showed differential expression between any two adjacent steps ($|\log_2$ fold-change$|>1.2$ corrected p-value < 0.01) (Additional file 2: Table S17) and motif enrichment in the clustered events. For each differentiation stage, the values shown are the minimum, maximum and midpoint value of expression, in $\log_{10}$(TPM+0.1) scale, across the three replicates for each gene. The dashed lines connect the midpoints for each factor at each stage. Both *MBNL2* and *MBNL3* showed significant expression decrease between stages 2 and 3, but the fold-change given by DESeq2 did not pass the used filter (Additional file 2: Table S17) (Methods).

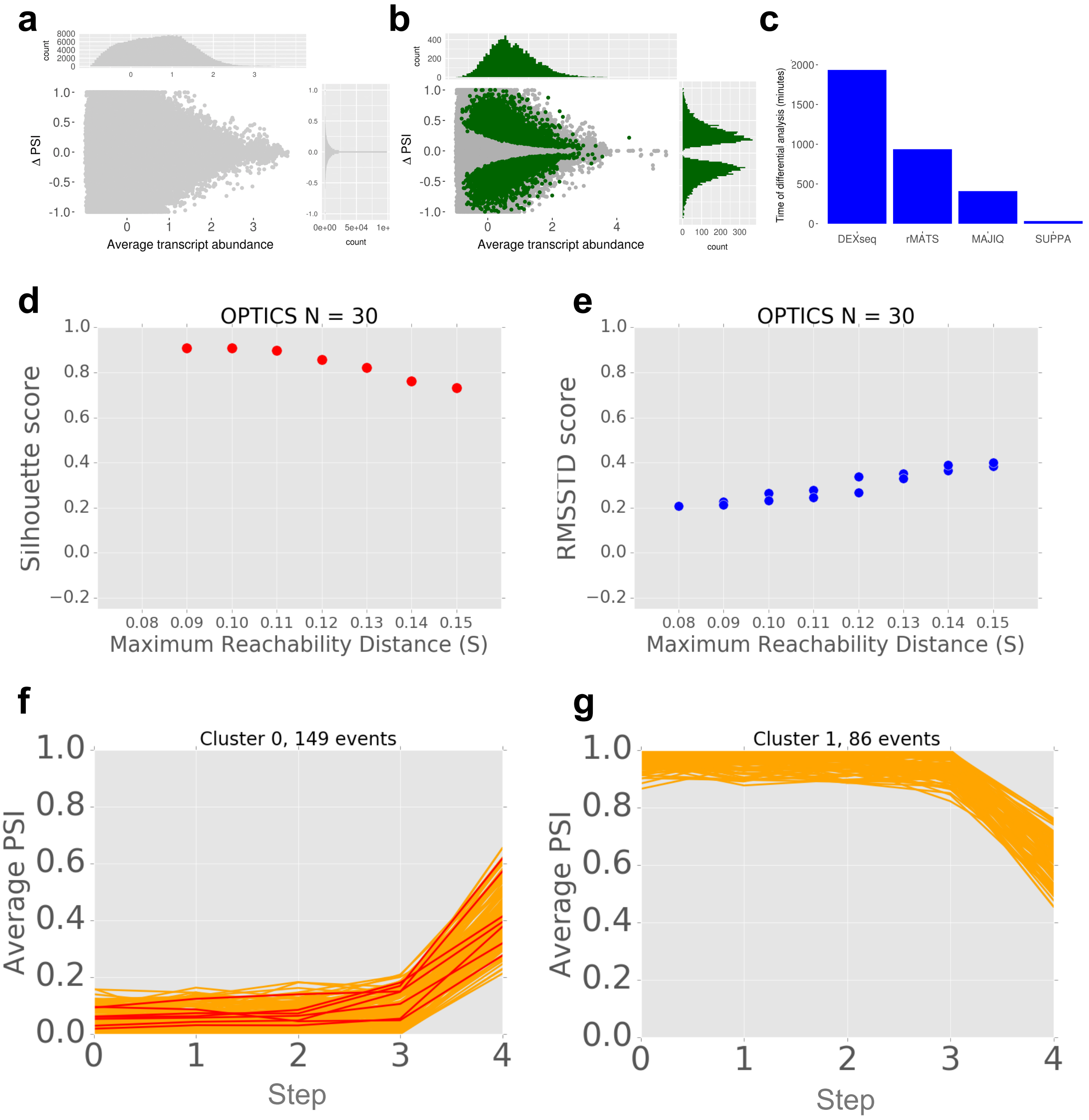
a
b
c
count
Δ PSI
Average transcript abundance
Time of differential analysis (minutes)
DEXseq
rMATS
MAJIQ
SUPPA
d
e
OPTICS N = 30
Silhouette score
RMSSTD score
Maximum Reachability Distance (S)
f
g
Cluster 0, 149 events
Cluster 1, 86 events
Average PSI
Step

Figure S6. SUPPA2 analysis of differential splicing across erythrocyte differentiation. (a) We show the between-replicate distribution of ΔPSI values as a function of the average abundance of the transcripts describing each event, using the initial (proerythroblast or proE) and last (orthochromatic erythroblasts or orthoE) steps of differentiation from [11]. The attached panels display the distribution of ΔPSI values along the x-axis (top panel) and along the y-axis (right panel). **(b)** Significant differentially spliced events (in green) between proE and orthoE steps superimposed on the background distribution from (a). The attached panels display the distribution of the significant ΔPSI values along the x-axis (top panel) and along the y-axis (right panel). **(c)** Time spent by SUPPA2, rMATS, MAJIQ and DEXSeq to analyze the differential splicing between proE and orthoE. **(d-e)** Density-based clustering was performed with OPTICS [9] for the events that changed splicing significantly between any two adjacent steps across erythroblast differentiation. Quality of the clustering was evaluated with SUPPA2 by measuring the silhouette score (d) and root mean square standard deviation (RMSSTD) (e) at different values of the maximum reachability distance (S), requiring a minimum number of 30 events per cluster and using Euclidean distance. Finally, **(f)** and **(g)** show the two clusters obtained for the optimal value of S=0.1. The plots represent the average PSI values (*y*-axis) per differentiation stage (*x*-axis) for the events in each cluster across conditions. Retained intron events are plotted in red over the rest of the events in orange.

References

1. Cui X, Churchill GA. Statistical tests for differential expression in cDNA microarray experiments. Genome Biol. [Internet]. 2003;4:210. Available from: http://www.ncbi.nlm.nih.gov/pubmed/12702200

2. Yang YH, Dudoit S, Luu P, Lin DM, Peng V, Ngai J, et al. Normalization for cDNA microarray data: a robust composite method addressing single and multiple slide systematic variation. Nucleic Acids Res. [Internet]. 2002;30:e15. Available from: http://www.ncbi.nlm.nih.gov/pubmed/11842121

3. Best A, James K, Dalgliesh C, Hong E, Kheirolahi-Kouhestani M, Curk T, et al. Human Tra2 proteins jointly control a CHEK1 splicing switch among alternative and constitutive target exons. Nat. Commun. [Internet]. 2014;5:4760. Available from: http://www.ncbi.nlm.nih.gov/pubmed/25208576

4. Patro R, Mount SM, Kingsford C. Sailfish enables alignment-free isoform quantification from RNA-seq reads using lightweight algorithms. Nat. Biotechnol. [Internet]. 2014;32:462–4. Available from: http://www.ncbi.nlm.nih.gov/pubmed/24752080

5. Vaquero-Garcia J, Barrera A, Gazzara MR, González-Vallinas J, Lahens NF, Hogenesch JB, et al. A new view of transcriptome complexity and regulation through the lens of local splicing variations. Elife [Internet]. 2016;5:e11752. Available from: http://www.ncbi.nlm.nih.gov/pubmed/26829591

6. Zhang R, Lahens NF, Ballance HI, Hughes ME, Hogenesch JB. A circadian gene expression atlas in mammals: implications for biology and medicine. Proc. Natl. Acad. Sci. U. S. A. [Internet]. 2014;111:16219–24. Available from: http://www.ncbi.nlm.nih.gov/pubmed/25349387

7. Cole BS, Tapescu I, Allon SJ, Mallory MJ, Qiu J, Lake RJ, et al. Global analysis of physical and functional RNA targets of hnRNP L reveals distinct sequence and epigenetic features of repressed and enhanced exons. RNA [Internet]. 2015;21:2053–66. Available from: http://www.ncbi.nlm.nih.gov/pubmed/26437669

8. Busskamp V, Lewis NE, Guye P, Ng AHM, Shipman SL, Byrne SM, et al. Rapid neurogenesis through transcriptional activation in human stem cells. Mol. Syst. Biol. [Internet]. 2014;10:760. Available from: http://www.ncbi.nlm.nih.gov/pubmed/25403753

9. Ankerst M, Breunig MM, Kriegel H, Sander J. OPTICS: Ordering Points To Identify the Clustering Structure. ACM Sigmod Rec. 1999;28:49–60.

10. Ester M, Kriegel HP, Sander J, Xu X. A Density-Based Algorithm for Discovering Clusters in Large Spatial Databases with Noise. Proc. 2nd Int. Conf. Knowl. Discov. Data Min. [Internet]. 1996. p. 226–31. Available from: https://www.aaai.org/Papers/KDD/1996/KDD96-037.pdf

11. Pimentel H, Parra M, Gee S, Ghanem D, An X, Li J, et al. A dynamic alternative splicing program regulates gene expression during terminal erythropoiesis. Nucleic Acids Res. [Internet]. 2014;42:4031–42. Available from: http://www.ncbi.nlm.nih.gov/pubmed/24442673
