# Supplementary material for: SUPPA2: fast, accurate, and uncertainty-aware differential splicing analysis across multiple conditions
Source: Genome Biol. 2018 Mar 23;19:40. doi: 10.1186/s13059-018-1417-1 (PMC5866513; doi:10.1186/s13059-018-1417-1)
Supplement: Supplementary file 3 — Supplementary methods. (PDF 315 kb) [file 13059_2018_1417_MOESM3_ESM.pdf]

Supplementary Material

SUPPA2 provides fast, accurate, and uncertainty-aware differential splicing analysis across multiple conditions

Juan L. Trincado[1,*], Juan C. Entizne[2,*], Gerald Hysenaj[3], Babita Singh[1], Miha Skalic[1], David J. Elliott[3], Eduardo Eyras[1,4]

[1]Pompeu Fabra University, E08003, Barcelona, Spain

[2]University of Dundee, Invergowrie, Dundee DD2 5DA, UK.

[3]Institute of Genetic Medicine, Newcastle University, Central Parkway, Newcastle NE1 3BZ, UK.

[4]ICREA, E08010, Barcelona, Spain

* equal contribution

corresponding author: eduardo.eyras@upf.edu

Software and datasets

SUPPA2 is available at https://github.com/comprna/SUPPA
Commands and datasets used in this work are available at
https://github.com/comprna/SUPPA_supplementary_data

SUPPA2

As SUPPA [1], SUPPA2 generates events from a gene annotation and keeps track of the transcripts that contribute to each event [1]. An alternative splicing event is the representation of a local variation of the exon-intron structure that is defined by the transcripts that cover that genic region, and is represented in binary form, e.g. inclusion and skipping of a cassette exon. Accordingly, an event is characterized in terms of the sets of transcripts that describe either form of the event, which can be denoted as F_1 and F_2. For instance, for the exon cassette event (SE), F_1 represents the transcripts that include the exon, whereas F_2 represents the transcripts that skip the exon. The inclusion value, proportion spliced-in (PSI), or percent spliced-in when scaled between 0 and 100%, of an event is defined as the ratio of the abundance of transcripts that include one form of the

event, F_1, over the abundance of the transcripts that contain either form of the event [2]. That is, given the abundances for the transcripts isoforms in transcript per million units (TPM) [3], which we denote as TPM_k, SUPPA2 calculates the PSI for an event as follows:

$$PSI = \frac{\sum_{k \in F_1} TPM_k}{\sum_{j \in F_1 \cup F_2} TPM_j}$$

SUPPA2 generates different alternative splicing event types from an input annotation file in GTF format: exon skipping (SE), alternative 5' and 3' splice-sites (A5/A3), mutually exclusive exons (MX), intron retention (RI), and alternative first and last exons (AF/AL). The PSI value for an event is calculated with respect to one of the two forms of the event. Further details on how the events are defined and PSIs calculated are given at https://github.com/comprna/SUPPA.

As the uncertainty on the PSI value increases at low expression, SUPPA2 allows fixing a lower bound *b* for the total expression (denominator in the formula above) for the calculation of the PSI:

$$\sum_{j \in F_1 \cup F_2} TPM_j > b$$

The default is no filter, i.e. *b=0.* Additionally, SUPPA2 is agnostic of the actual methodology for quantifying transcripts. For the analyses of this article we have used Salmon [4] to quantify transcripts from the Ensembl annotation. However, other methods show similar results [1].

SUPPA2 Differential splicing

Differential splicing is calculated from the files of event PSI values and transcript abundances in transcripts per million (TPM) units. Given two conditions, with two or more replicates per condition, the ΔPSI is calculated for each event as the difference of mean values between conditions, and this value is compared to the ΔPSI values between replicates of the same condition as a function of the average transcript abundance. For each event, the average transcript abundance between replicates is calculated in $\log_{10}$(TPM) units. Given $TPM_{a,r}$ the abundances of the transcripts *a=1,..,n* describing a given event in each replicate sample *r,* the average transcript abundance associated to this event is calculating by first adding the total abundance of the transcripts per replicate and then averaging over all replicates $|R_c|$:

$$E_{rep} = \frac{1}{|R_c|} \sum_{r \in R_c} \log_{10}\left(\sum_a TPM_{a,r} \right)$$

where $r=1,..,|R_c|$ runs over the replicates, and a runs over the transcripts that describe the event. For instance, for an exon cassette event, these would be the transcripts that describe the inclusion or the exclusion forms. Similarly, for each event we define the average transcript abundance between conditions adding up the TPM values for all transcripts describing the event in each sample, and then averaging in log10 scale between replicates and conditions:

$$E_{cond} = \frac{1}{2}\sum_{c=1,2}\frac{1}{|R_c|}\sum_{r\in R_c}\log_{10}\left(\sum_a TPM_{a,r,c}\right)$$

where $r=1,..,|R_c|$ runs over the replicates in each condition $c=1,2$ for each transcript a describing the event, and $TPM_{a,r,c}$ is the abundance of transcript a in the replicate r in the condition c. The ΔPSI value between a pair of replicates is calculated as the difference of PSI values, whereas the ΔPSI value between conditions is calculated as the difference of the means of the two conditions.

Given the observed ΔPSI between conditions and E_{cond} for an event, the significance is calculated by comparing with the ΔPSI distribution between replicates for events with E_{rep} values similar to the observed E_{cond}. These are obtained by selecting the closest value E^*_{rep} from all points i from the background distribution:

$$E^*_{rep} = \min_i\left\{\left|E_{i,rep} - E_{cond}\right|\right\}$$

using binary search along the list of values and selecting a fixed number of events on either side of E^*_{rep}. By default, we use 1000 values around the E^*_{rep} value, including it. The selected window of events defines an empirical cumulative density function (ECDF) over |ΔPSI| values from which a p-value is calculated:

$$p = \left(1 - ECDF\left(|\Delta PSI|\right)\right)/2$$

With this we are assuming that the background distribution is symmetric. That is, the distribution of ΔPSI values between replicates is centered around zero and with similar frequencies of positive and negative values. Additionally, there is an option to avoid testing events with |ΔPSI| value between conditions below certain threshold, which speeds up the analysis.

SUPPA2 also includes the possibility to perform a classical statistical test between conditions when there are sufficient replicates (>10). In this case, the Wilcoxon rank-sum test, or signed-rank test if the data is paired, is applied per event to test the significance, as previously applied in [5]. The change of the splicing event (ΔPSI) is again defined as the difference of the mean PSI values in each condition. For many replicates per condition, the

classical test is much faster than the empirical method. However, the classical test does not take into account the variability across replicates. For both statistical tests, SUPPA2 includes an option to correct for multiple testing across all events from the same gene, as they may not be independent from each other. Multiple test correction is performed with the Benjamini-Hochberg method and the false discovery rate (FDR) cut-off can also be read as an input parameter.

Comparisons can be performed between two conditions or between multiple conditions. For multiple conditions, the default is to compare pairwise adjacent conditions in an ordered list specific in the command line. Alternatively, there is also the option to perform all pairwise comparisons across the multiple conditions. See https://github.com/comprna/SUPPA for a description of the commands and options.

As described above, for the calculation of PSI values, a filter on the minimum total expression of transcripts describing the event can be used, and has default 0. Similarly, a filter can be also applied at the step of differential splicing calculation. The filter is applied as follows: given a value X for the lower-bound of the expression, which can provided in the command line, we only keep events that have the average expression in each condition larger or equal that this expression in $\log_{10}$ scale:

$$E_{rep} = \frac{1}{|R_c|}\sum_{r\in R_c} \log_{10}\left(\sum_a TPM_{a,r}\right) \geq \log_{10}(X)$$

where this constrained is applied to both conditions. As the differential splicing is calculated in terms of the average expression in both conditions (see equation for E_{cond} above), the filtering implies $E_{cond} > log_{10}(X)$. However, note that the double condition that we are applying is stronger.

Experimental datasets

We have analyzed RNA sequencing (RNA-seq) data from knockdown of TRA2A+TRA2B and controls in MDA-MB-231 cells with 3 replicates per condition [6] (GSE59335). RT-PCR data for these conditions was obtained from [6] and from new experiments (this work) (RT-PCR performed in triplicates). We also analyzed data from Cerebellum and Liver mouse tissues covering 8 different time points from 2 full circadian cycles [7] (GSE54651). For the comparison with the RT-PCR results from [8] (validated with three replicates each) we compared CT28, CT40 and CT52 in Cerebellum with the same time points in Liver. We also analyzed the differential splicing between stimulated and unstimulated Jurkat T-cells [9]

(SRP059357) to perform the comparison with RT-PCR results from [8]. For this comparison, as these the 54 RT-PCR validated events were not tested in triplicates, we only used the 30 events that had |ΔPSI|>0.05 from the RT-PCR. We further used RNA-seq samples from a 4-day time-course, 3 replicates each, for the differentiation of human iPS cells into bipolar neurons [10] (GSE60548). In this case we focused on differentially spliced events of exon-cassette type that changed significantly and with at least |ΔPSI|>0.2 between adjacent stages. Additionally, we analyzed RNA-seq from differentiating human erythroblasts [11] (GSE53635), which is composed of 5 different differentiation steps with 3 replicates per condition.

Simulated datasets

We used the quantification of the RefSeq transcripts with RSEM [3] for the 3 control samples from [6] (GSE59335) as theoretical abundances (in TPM units) and considered genes with only two isoforms containing an skipping exon (SE) or alternative 5'/3' splice-site (A5/A3). In addition, we restricted the genes for the benchmark to be associated to only 1 event and with absolute difference of relative abundance between the two isoforms greater than 0.2 given by:

$$\frac{|TPM_1 - TPM_2|}{TPM_1 + TPM_2} > 0.2$$

where *TPM1* and *TPM2* are the abundances of the only two transcripts in a gene in TPM (transcripts per million) units. For the positive set, we selected 277 SE events and 318 A5/A3 events and simulated differential splicing by exchanging the theoretical TPM values for these transcripts in a second condition in all three replicates, keeping the same theoretical abundances for the transcripts in all other genes. For the negative set, we selected an equal number of genes sampled from the entire range of values without exchanging their TPM values. These negative events are expected to have variability between conditions similar to the variability between biological replicates.

We used RSEM [3] to simulate sequencing reads for the 2 conditions, 3 replicates each, at various depths: 120, 60, 25, 10 and 5 millions of 100nt paired-end reads per sample, and at various read lengths: 100nt, 75nt, 50nt and 25nt, at depth 25M paired-end reads (Supp. Tables S1-S3). For the simulated data SUPPA2 was run using transcript quantification with Salmon [4] on the RefSeq annotation. Simulated reads were mapped to the genome using TopHat [12] and STAR [13]. The benchmarking analysis was run with both. All methods show similar behaviour for long read-length (~100nt) and high coverage (>25M paired-end reads). Results shown correspond to the analyses with TopHat.

For each method we calculated the events that can be considered observed or measured, i.e. the method recovers the coordinates of the event or regulated exon and provides a ΔPSI (SUPPA2, rMATS, MAJIQ) or log-fold change (for DEXSeq), regardless of whether the event is predicted as significant. From these recovered cases, we calculated the subset of true positives. A measured positive event was considered a true positive if it was statistically significant according to the statistical test performed by the method and had a ΔPSI change or log-fold change in the same direction as the simulated event, regardless of the size of this change. As a comparison, we also calculated true positives imposing in addition a cutoff $|\Delta PSI|>0.2$ for the predictions, but saw no or very little change in the results (Additional file 2: Table S3). For SUPPA2, rMATS and DEXSeq, an event was significant for a corrected p-value < 0.05. For MAJIQ an event was considered recovered if there was at least one LSV with one of the inclusion junctions of the event with a calculated ΔPSI value, and it was considered significant if the posterior for $|\Delta PSI|>0.1$ was > 0.95. Since all cassette events were defined from genes with only two transcripts, this definition of recovered event was unambiguous. That is, despite their very different descriptions, we could unambiguously match the results from SUPPA2, DEXSeq, rMATS and MAJIQ to the reference set of positive and negative cassette events.

Comparison with RT-PCR data

Transcripts from Ensembl (version 75 – without pseudogenes) were quantified using Salmon [4]. SUPPA2 was run using only transcripts with TPM > 0.1. Using transcript abundances from Sailfish [14] or Kallisto [15] produced similar results in the comparison to RT-PCR data (data not shown). RNA-seq reads were also mapped to the genome using the rMATS mapping pipeline, which runs TopHat [12]. All methods other than SUPPA2 were used with these mappings. rMATS was run using the junction and exon body reads (ReadsOnTargetAndJunctionCounts).

SUPPA2 events of type skipping exon (SE) were matched to the RT-PCR validated events in each dataset. We only considered events for which the middle exon matched exactly with the validated exon and the flanking exons of the event coincided with those on which RT-PCR primers were placed. Ambiguous matches were discarded. rMATS events were matched in a similar way. For MAJIQ, as ΔPSI values are given per junction but junctions can be duplicated in different local splicing variations (LSVs), we selected the inclusion junction compatible with the validated event that had the largest posterior probability if there were two, or the only compatible inclusion junction if only one was available. For DEXSeq, as it only describes exonic regions, we considered the exonic regions that matched exactly the regulated exon of the validated event.

Comparison between methods

The direct comparison between alternative splicing events measured by different methods is not straightforward as different methods usually have a very different representation of what an alternative splicing variation is. We decided to focus on events of type exon cassette (SE) and alternative 5' (A5) or 3' (A3) splice site, which are the most common types and are described by all methods. We first identified those events that were common to SUPPA2 and rMATS, as they describe SE and A5/A3 events in the same way. That is, we selected events measured by both, but not necessarily significant. As DEXSeq works with exonic regions that may not always correspond to a full exon, we selected the subset of SE events for which the middle exon from an SE event or a variable region from an A5/A3 event matched exactly a DEXSeq exonic region. From these cases we eliminated the redundant cases produced by events describing the same regulated exon but with different pairs of flanking splicing sites. This set was then compared to MAJIQ, which produces local splicing variations (LSVs). Each LSV is composed of multiple junctions, which is the element for which a ΔPSI value and a statistical test is produced, and the same junction can appear in more than one LSV with different ΔPSI and statistical test result. We thus compared directly the selected events common between SUPPA2, rMATS and DEXSeq with the MAJIQ junctions. For SE events, we selected from MAJIQ the inclusion junction instance compatible with the event and with the largest posterior probability for $|\Delta PSI|>0.1$. For A5/A3 events we selected from MAJIQ the junction instance that describes the form of the event that makes the intron shorter (which is the one for which SUPPA2 and rMATS give the PSI value) and with the largest posterior probability for $|\Delta PSI|>0.1$. We further kept only those events from genes that showed non-zero gene expression, calculated as the sum of transcript TPMs averaged across the control replicates. This yielded a set of 7116 SE events and 2924 A5/A3 events unambiguously measured by all four methods, and which we can compare directly for effect-size and significance.

Experimental validation

MDA-MB-231 breast cancer cells were cultured in DMEM media with 10% FBS and 1% penicillin/streptomycin until approximately 80% confluence was reached. Three biological replicates of Tra2 double knockdown were prepared using TRA2A and TRA2B targeting siRNAs (Ambion: s12749 and s26664). Control cells were transfected with scramble siRNA (Ambion Cat#: 4390843). Lipofectamine RNAiMAX was used for siRNA transfection following manufacturer's protocol. RNA was extracted using standard Trizol RNA extraction (Life Technologies) following manufacturer's instructions. cDNA was synthesized from total RNA using Superscript VILO cDNA synthesis kit (Invitrogen). GoTaq G2 DNA polymerase kit (Promega) was used to PCR-amplify the target exons using primers in flanking exons (primers available in Supp. Table S14). Splicing profiles were quantified using the Qiaxcel

capillary electrophoresis system (Qiagen). Student t-test was used to test the significance of the splice changes between the two conditions using the 3 replicates per condition.

Clustering analysis

SUPPA2 currently implements two density-based clustering methods: DBSCAN [16] and OPTICS [17]. Density-based clustering has the advantage that one does not need to specify the expected number of clusters, and the choice between the two methods depends mainly on the computational resources and the amount of data. Both methods use the vectors of mean PSI values per event and require as input the minimum number of events in a cluster (N), which indicates the expected sizes of the possible regulatory modules; and the minimum separation between clusters (S), which approximates the average total difference of PSI at which two events are considered to behave similarly across conditions. Additionally, DBSCAN needs the maximum distance to consider two events as cluster partners (D), which OPTICS calculates through an optimization procedure. DBSCAN allows performing simple and fast data partitioning but has the drawback of being sensitive to the input parameters. On the other hand, OPTICS, which can be seen as a generalization of DBSCAN, explores the possible maximum values for D beyond which clustering quality drops. OPTICS can thus potentially produce better clustering results since it is not limited to a fixed radius of clustering, but it is penalized by a greater computational cost. Clustering is only performed with events that change significantly in at least one pair of conditions. Additionally, there is the possibility to impose restrictions on the |ΔPSI| and significance p-value of the events to be clustered. Cluster qualities are reported using the silhouette score [18], which indicates how well the events are assigned to clusters; and the root mean square standard deviation (RMSSTD), which measures the homogeneity of each cluster. Additionally, the number of events in each cluster and percentage of events in clusters are also reported. Three different distance metrics can be currently used: Euclidean, Manhattan and Cosine.

CLIP analysis

Significant CLIP signals for TRA2B in MDA-MB-231 cells were obtained from [6] (GSE59335). Bedgraph files were converted to bed using pyicoteo [19] and only CLIP peaks with more than 5 reads were kept. We then calculated which of the 7116 cassette events had a CLIP cluster within 100nt from the event coordinates, i.e. from 100nt upstream of the 5’ splice-site of the 5’ exon to 100nt downstream of the 3’ splice-site of the 3’ exon. For each method the 7116 events were split according to whether they were found as significant or not, and whether they had CLIP clusters or not, and a 2x2 contingency table was built to

perform a Fisher's exact test. Results are given in Supp. Table S13.

Motif analysis

Motif enrichment analysis on the regulated events was performed using the tool MoSEA (https://github.com/comprna/MoSEA) [5]. This tool performs motif enrichment analysis between two sets of sequences: a set of interest and corresponding controls. Motifs can be used in terms of position weight matrices (PWMs), lists of k-mers associated to labels, or it can be used to do an unbiased k-mer search. For PWMs, it uses FIMO [20] to scan the motifs from a list of PWMs. We used PWMs from RNAcompete [21] in the regions of 200nt upstream, exon and 200nt downstream in exon-cassette events using as cut-off p-value < 0.001. To this set of motifs we added the 6-mers that were detected before enriched in frequency and in position upstream of SRRM4-bound microexons [22] (TGCTGC, GCTGCC). MoSEA performs motif enrichment analysis by comparing the frequency of regions in differentially spliced events with a specific motif label (e.g. events with matches for an CELF4 motif) with 100 random subsamples of the same number of cases from non-differentially spliced exon-cassette events, using the same region type, and controlling for G+C content, and for sequence length in the case of exons. MoSEA calculates an enrichment Z-score per motif label and region using the observed frequency and the frequencies in the 100 random control sets. The regions considered for exon cassette events are the exon itself, and 200nt upstream and downstream (see [5] for the definition of the regions for all alternative splicing event types). We only considered motifs with a minimum frequency of 20%, i.e. they occurred in 20% of the regulated events for a given specific region. Figure 4 only displays those enriched motifs that correspond to RBPs that are also differentially expressed between any two adjacent differentiation steps. The software for motif enrichment analysis is available at https://github.com/comprna/MOSEA.

Differential expression analysis

To perform differential expression analysis between adjacent stages of neuronal differentiation [10] we considered the transcript read counts estimated with Salmon [4] for the Ensembl annotation (version 75), and used tximport [23] to group transcript read counts per gene with scaling by transcript length and library size (lengthScaledTPM). We then used DESeq2 [24] with these read-counts per gene. Genes were considered differentially expressed for a log fold-change of $|logFC|>1.2$ and a corrected p-value < 0.01. The results for RBPs are available in Additional file 2: Table S17.

References

1. Alamancos GP, Pagés A, Trincado JL, Bellora N, Eyras E. Leveraging transcript quantification for fast computation of alternative splicing profiles. RNA. 2015;21:1521–31.

2. Venables JP, Klinck R, Bramard A, Inkel L, Dufresne-Martin G, Koh C, et al. Identification of alternative splicing markers for breast cancer. Cancer Res. [Internet]. 2008;68:9525–31. Available from: http://www.ncbi.nlm.nih.gov/pubmed/19010929

3. Li B, Dewey CN. RSEM: accurate transcript quantification from RNA-Seq data with or without a reference genome. BMC Bioinformatics [Internet]. 2011;12:323. Available from: http://www.ncbi.nlm.nih.gov/pubmed/21816040

4. Patro R, Duggal G, Love MI, Irizarry RA, Kingsford C. Salmon provides fast and bias-aware quantification of transcript expression. Nat. Methods [Internet]. 2017; Available from: http://www.ncbi.nlm.nih.gov/pubmed/28263959

5. Sebestyén E, Singh B, Miñana B, Pagès A, Mateo F, Pujana MA, et al. Large-scale analysis of genome and transcriptome alterations in multiple tumors unveils novel cancer-relevant splicing networks. Genome Res. [Internet]. 2016;26:732–44. Available from: http://www.ncbi.nlm.nih.gov/pubmed/27197215

6. Best A, James K, Dalgliesh C, Hong E, Kheirolahi-Kouhestani M, Curk T, et al. Human Tra2 proteins jointly control a CHEK1 splicing switch among alternative and constitutive target exons. Nat. Commun. [Internet]. 2014;5:4760. Available from: http://www.ncbi.nlm.nih.gov/pubmed/25208576

7. Zhang R, Lahens NF, Ballance HI, Hughes ME, Hogenesch JB. A circadian gene expression atlas in mammals: implications for biology and medicine. Proc. Natl. Acad. Sci. U. S. A. [Internet]. 2014;111:16219–24. Available from: http://www.ncbi.nlm.nih.gov/pubmed/25349387

8. Vaquero-Garcia J, Barrera A, Gazzara MR, González-Vallinas J, Lahens NF, Hogenesch JB, et al. A new view of transcriptome complexity and regulation through the lens of local splicing variations. Elife [Internet]. 2016;5:e11752. Available from: http://www.ncbi.nlm.nih.gov/pubmed/26829591

9. Cole BS, Tapescu I, Allon SJ, Mallory MJ, Qiu J, Lake RJ, et al. Global analysis of physical and functional RNA targets of hnRNP L reveals distinct sequence and epigenetic features of repressed and enhanced exons. RNA [Internet]. 2015;21:2053–66. Available from: http://www.ncbi.nlm.nih.gov/pubmed/26437669

10. Busskamp V, Lewis NE, Guye P, Ng AHM, Shipman SL, Byrne SM, et al. Rapid neurogenesis through transcriptional activation in human stem cells. Mol. Syst. Biol. [Internet]. 2014;10:760. Available from: http://www.ncbi.nlm.nih.gov/pubmed/25403753

11. Pimentel H, Parra M, Gee S, Ghanem D, An X, Li J, et al. A dynamic alternative splicing program regulates gene expression during terminal erythropoiesis. Nucleic Acids Res. [Internet]. 2014;42:4031–42. Available from: http://www.ncbi.nlm.nih.gov/pubmed/24442673

12. Kim D, Pertea G, Trapnell C, Pimentel H, Kelley R, Salzberg SL. TopHat2: accurate alignment of transcriptomes in the presence of insertions, deletions and gene fusions. Genome Biol. [Internet]. 2013;14:R36. Available from: http://www.ncbi.nlm.nih.gov/pubmed/23618408

13. Dobin A, Davis CA, Schlesinger F, Drenkow J, Zaleski C, Jha S, et al. STAR: Ultrafast

universal RNA-seq aligner. Bioinformatics. 2013;29:15–21.

14. Patro R, Mount SM, Kingsford C. Sailfish enables alignment-free isoform quantification from RNA-seq reads using lightweight algorithms. Nat. Biotechnol. [Internet]. 2014;32:462–4. Available from: http://www.ncbi.nlm.nih.gov/pubmed/24752080

15. Bray NL, Pimentel H, Melsted P, Pachter L. Near-optimal probabilistic RNA-seq quantification. Nat. Biotechnol. [Internet]. 2016;34:525–7. Available from: http://www.ncbi.nlm.nih.gov/pubmed/27043002

16. Ester M, Kriegel HP, Sander J, Xu X. A Density-Based Algorithm for Discovering Clusters in Large Spatial Databases with Noise. Proc. 2nd Int. Conf. Knowl. Discov. Data Min. [Internet]. 1996. p. 226–31. Available from: https://www.aaai.org/Papers/KDD/1996/KDD96-037.pdf

17. Ankerst M, Breunig MM, Kriegel H, Sander J. OPTICS: Ordering Points To Identify the Clustering Structure. ACM Sigmod Rec. 1999;28:49–60.

18. Rousseeuw PJ. Silhouettes: A graphical aid to the interpretation and validation of cluster analysis. J. Comput. Appl. Math. North-Holland; 1987;20:53–65.

19. Althammer S, González-vallinas J, Ballaré C, Beato M, Eyras E. Pyicos: A versatile toolkit for the analysis of high-throughput sequencing data. Bioinformatics. 2011;27.

20. Bailey TL, Boden M, Buske FA, Frith M, Grant CE, Clementi L, et al. MEME SUITE: tools for motif discovery and searching. Nucleic Acids Res. 2009;37:W202–8.

21. Ray D, Kazan H, Cook KB, Weirauch MT, Najafabadi HS, Li X, et al. A compendium of RNA-binding motifs for decoding gene regulation. Nature [Internet]. 2013;499:172–7. Available from: http://www.ncbi.nlm.nih.gov/pubmed/23846655

22. Raj B, Irimia M, Braunschweig U, Sterne-Weiler T, O'Hanlon D, Lin ZY, et al. A global regulatory mechanism for activating an exon network required for neurogenesis. Mol. Cell. 2014;56:90–103.

23. Soneson C, Love MI, Robinson MD. Differential analyses for RNA-seq: transcript-level estimates improve gene-level inferences. F1000Research [Internet]. 2015;4:1521. Available from: http://www.ncbi.nlm.nih.gov/pubmed/26925227

24. Love MI, Huber W, Anders S. Moderated estimation of fold change and dispersion for RNA-seq data with DESeq2. Genome Biol. [Internet]. 2014;15:550. Available from: http://www.ncbi.nlm.nih.gov/pubmed/25516281
